# Zebrafish Get Connected: Investigating Neurotransmission Targets and Alterations in Chemical Toxicity

**DOI:** 10.3390/toxics4030019

**Published:** 2016-08-27

**Authors:** Katharine A. Horzmann, Jennifer L. Freeman

**Affiliations:** School of Health Sciences, Purdue University, West Lafayette, IN 47907, USA; khorzmann@purdue.edu

**Keywords:** acetylcholine, dopamine, GABA, glutamate, neurotoxicology, neurotransmission, neurotransmitters, norepinephrine, serotonin, zebrafish

## Abstract

Neurotransmission is the basis of neuronal communication and is critical for normal brain development, behavior, learning, and memory. Exposure to drugs and chemicals can alter neurotransmission, often through unknown pathways and mechanisms. The zebrafish (*Danio rerio*) model system is increasingly being used to study the brain and chemical neurotoxicity. In this review, the major neurotransmitter systems, including glutamate, GABA, dopamine, norepinephrine, serotonin, acetylcholine, histamine, and glutamate are surveyed and pathways of synthesis, transport, metabolism, and action are examined. Differences between human and zebrafish neurochemical pathways are highlighted. We also review techniques for evaluating neurological function, including the measurement of neurotransmitter levels, assessment of gene expression through transcriptomic analysis, and the recording of neurobehavior. Finally examples of chemical toxicity studies evaluating alterations in neurotransmitter systems in the zebrafish model are reviewed.

## 1. Introduction

Neurotransmission is the basis of neuronal communication and is critical for normal brain development, behavior, learning and memory, and even maintenance of life. The nervous system is exceedingly complex, and many enzymes, transporters, and receptors all work in concert to maintain these functions. Neurotransmission can be altered by exposure to drugs, pharmaceuticals, chemotherapeutic agents, radiation, food additives, and environmental toxicants including pesticides and heavy metals ([1,2] and as reviewed by [3,4,5,6]). Alterations in neurotransmission have been linked to a number of diseases including movement disorders, neuropsychiatric disorders, and depression (reviewed by [7,8,9]).

The zebrafish (*Danio rerio*) research model is increasingly being used in neurotoxicity studies (as reviewed by [3,10,11,12,13]). Zebrafish share the common neurotransmitter pathways with mammals and have similar neuroanatomy in many areas such as the spinal cord, hindbrain and retina, but as the brain develops by eversion rather than inversion, some classical regions of the mammalian brain, such as the hippocampus, amygdala, and substantia nigra, are not present as such in zebrafish. The function of these areas appears to be maintained elsewhere in the brain, allowing functional comparisons between zebrafish and mammals [14]. As an additional resource, Mueller and Wullimann have recently published a second edition of their “Atlas of Early Zebrafish Brain Development”, which characterizes neuronal development and provides excellent figure panels for neuroanatomy [15].

Although the neurochemistry of zebrafish has been reviewed previously [11,16], this review will specifically focus on reviewing the application of the zebrafish in chemical toxicology studies investigating adverse impacts to neurotransmitter systems. In addition, this review includes new research findings published since the last reviews on zebrafish neurochemistry, updated terminology, and aims to serve as a reference for the major neurotransmitter systems.

## 2. Zebrafish as a Biomedical Model

The zebrafish is a well-recognized biomedical research model. Zebrafish have been utilized in many scientific disciplines including developmental biology, drug discovery, pharmacology, genetics, and toxicology (reviewed in [17,18,19,20]). The zebrafish has many strengths as a research model. The adults are small (up to 3 cm), and large colonies can be easily maintained with basic husbandry. Zebrafish have a short generational interval, with sexual maturity beginning at 3–4 months post fertilization (mpf). Once mature, a single breeding pair of zebrafish can produce 100–200 fertilized embryos per spawning. The embryos develop ex utero, are nearly transparent, and are easily manipulated for developmental studies [21,22]. Zebrafish are also ideal for genetic manipulation, with ex utero fertilization allowing for the production of haploid embryos [23].

The zebrafish genome has been mapped and approximately 70%–80% of zebrafish genes share homology with the human genome, and 84% of genes associated with disease in humans are also present in zebrafish [24,25]. Furthermore, metabolic pathways are highly conserved between zebrafish and mammals, making zebrafish well suited to mechanism focused research [18].

The early teleost had a whole-genome duplication event approximately 320–350 million years ago [26,27]. Although nearly half of these gene duplicates are thought to have been lost within the first 75 million years, it is hypothesized that the remaining genes may have undergone a process of subfunctionalization or neofunctionalization [28,29]. Genes with a single copy in zebrafish are referred to as being orthologs to human genes if they share a common gene origin, while genes with two copies in zebrafish are termed paralogous and may have sub- or neofunctionalization [30]. The teleost whole-genome duplication event has important implications for the study of gene functions in biological pathways, including the neurotransmitter systems where two paralogs may have divergent functions.

## 3. Review of Neurotransmitter Systems

Zebrafish share the common neurotransmitter systems with other vertebrates, and therefore can serve as a model system for neurotoxicity. Although there are many more similarities than not, there are some noteworthy differences between mammals and zebrafish within the neurotransmitter systems, mainly in the number and name of genes encoding proteins, as a result of the teleost gene duplication event. In general, the synthesis and metabolism pathways are shared between the brain of mammals and teleosts. The major shared neurotransmitter systems are reviewed and differences in relevant genes, anatomy, and physiology are highlighted below.

### 3.1. Glutamate

Glutamate is the most common neurotransmitter in the mammalian and teleost brain, with an expected 80%–90% of mammalian synapses using glutamate as the neurotransmitter [31]. Glutamate is the primary excitatory neurotransmitter with functions associated with neurodevelopment, learning and memory, and general cognition as well as neurodegenerative diseases and pathologic conditions such as epilepsy, amnesia, cerebral ischemia, motor neuron diseases, pain, and psychosis [32,33]. Glutamate is also associated with synaptic plasticity and, depending on the receptor activated, may act to modulate neural impulses received by the postsynaptic neuron rather than excite [34,35].

Glutamate, an amino acid, is a member of multiple metabolic pathways including the tricarboxylic acid (TCA) cycle and is a precursor of many biologically important molecules including amino acids l-proline and l-arginine as well as the neurotransmitter γ-aminobutyric acid (GABA) and glutathione [33,36]. Most of the glutamate within the brain is produced from α-ketoglutarate, an intermediate of the TCA cycle. α-ketoglutarate is transaminated with another amino group, usually from aspartate, to form glutamate. This transamination is typically performed by aspartate aminotransferase, an enzymatic protein encoded by the glutamic-oxaloacetic transaminase 1 (*GOT1*) gene; however, other aminotransferases such as glutamic-pyruvate transaminases (GPT) can also produce glutamate [36].

Within neurons, the majority of glutamate is stored in synaptic vesicles. In mammals, the solute carrier (SLC) family SLC17 members SLC17A7, SLC17A6, and SLC17A8 (also known as the vesicular glutamate transporter family (VGLUT) 1, 2, 3, respectively) are responsible for transporting glutamate into synaptic vesicles, although only SLC17A7 and SLC17A6 are found in glutamatergic neurons [37]. Once released into the synaptic cleft, glutamate binds to receptors on postsynaptic neurons and adjacent glial cells. Most glutamate receptors are located on the dendritic spines of the postsynaptic neurons [38].

Glutamate receptors fall into two categories, ionotropic and metabotropic. Ionotropic receptors act by opening a cation channel after binding to the target and are thus part of the ligand-gated ion channel superfamily. The ligand-gated ion channel superfamily includes the ionotropic glutamate receptors, GABA_A_ receptors, 5-hydroxytryptamine 3 receptor (5-HT3), nicotinic acetylcholine receptors, and glycine receptors as reviewed by Collingridge et al. and Keramidas et al. [39,40]. The superfamily shares a common structure, with each channel being composed of 5 identical or homologous subunits surrounding the central pore [41]. Each subunit has an extensive extracellular, hydrophilic *N*-terminus, 4 transmembrane domains that form the ion channel, and an extracellular C-terminus. The superfamily can be divided into subfamilies which include the ionotropic glutamate receptors, the cys-loop receptor family, and ATP-gated channels. As reviewed by Connolly and Wafford [42] and Kozuska and Paulsen [43], the cys-loop receptor family is characterized by a loop on the *N*-terminus formed by a disulfide bond between two cysteines and includes the GABA_A_, 5-HT3, nicotinic acetylcholine, and glycine receptors. Each class of ligand-gated ion channels can be further divided based on if the channel is anion or cation specific, with glycine and GABA_A_ receptors being anionic and 5-HT3 and nicotinic acetylcholine receptors being cationic for example [40].

Metabotropic receptors act through second messenger systems. The term metabotropic is typically applied to a family of glutamate receptors that are G-protein-coupled receptors; however, GABA_B_ receptors, catecholamine receptors, all serotonin receptors except for 5-HT_3_, muscarinic acetylcholine receptors, and histamine receptors also act through second messenger systems and can be classified as metabotropic receptors due to their activation of G proteins and a variety of intracellular signaling cascades upon target binding [44,45,46]. As reviewed by Katritch et al., G-protein-coupled receptors have a shared structure with seven transmembrane domains and represent the largest superfamily of proteins [47]. A full description of G-protein-coupled receptors is beyond the scope of this review, but the topic has been extensively reviewed previously [48,49] and is commonly found in textbooks.

The functions, pharmacology, and mechanisms of the glutamate receptor types have been extensively reviewed [33,34,35,37,50,51,52]; key information is summarized here.

Ionotropic glutamate receptors are grouped into three classes: *N*-methyl d-aspartate (NMDA), α-amino-3-hydroxy-5-methyl-4-isoxazole propionic acid (AMPA), and kainite (KA). The classes were named according to selective agonists and each class is made up of multiple gene families which code for individual receptor subunits. The NMDA class receptor has seven genes which code for glutamate ionotropic receptor NMDA type subunits (GRIN, also known as glutamatergic ionotropic NDMA type; GLuNs): GRIN1, GRIN2A, GRIN2B, GRIN2C, GRIN2D, GRIN3A, and GRIN3B. AMPA receptors are made up of the glutamate ionotropic receptor AMPA type subunits (GRIA, also known as glutamatergic ionotropic AMPA type; GluA), GRIA1, GRIA2, GRIA3, and GRIA4, while glutamate ionotropic receptor kainate type subunits (GRIK, also known as glutamatergic ionotropic kainate type receptors; GluKs) fall into 5 families, GRIK1, GRIK2, GRIK3, GRIK4, and GRIK5 [52,53]. Upon binding glutamate, AMPA and KA receptors rapidly open ion channels that favor sodium conductance, while NMDA channels favor calcium conductance and have slower kinetics [51,52].

Metabotropic receptors are organized into three classes or groups, Group I, Group II, and Group III receptors. Group I receptors include GRM1 and GRM5. Group I receptors classically are coupled to G_q_/G_11_ proteins that activate inositol triphosphate (IP3) second messenger signaling and increase intracellular calcium levels, leading to postsynaptic depolarization [34]. Additional signaling pathways have also been recognized, with Group I receptors also acting through other G_q_ proteins, G_i/o_ proteins, G_s_ proteins, and independent of G proteins to activate alternative pathways. Downstream targets include phospholipase D, and protein kinase pathways such as Jun kinase, the mitogen-activated protein kinase/extracellular receptor kinase (MAPK/ERK) pathway, and the mammalian target of rapamycin (MTOR)/p70 S6 kinase pathway [54,55,56,57]. Group II receptors include the GRM2 and GRM3 subtypes and couple predominantly through G_i_/G_o_ proteins. These proteins act by decreasing 3′,5′-cyclic adenosine monophosphate (cAMP) levels through the inhibition of adenylyl cyclase, resulting in a hyperpolarization of the postsynaptic membranes [34]. Group III receptors are the GRM4, GRM6, GRM7, and GRM8 families and they also act by inhibiting adenylyl cyclase and decreasing intracellular cAMP levels. Group II and Group III members can also act through alternative signaling, similar to Group I receptors [50,58,59].

Glutamate is also taken up into glia and neurons via the SLC1A family of genes, also known as the high affinity excitatory amino acid transporters (EAATs). This gene family regulates the levels of extracellular glutamate to prevent excitotoxicity. In astrocytes and oligodendroglia, glutamate can be used in the TCA cycle for the production of energy, used for protein synthesis, or cycled back to glutamine. Glutamate-ammonia ligase (GLUL; glutamine synthetase) causes waste ammonia to react with glutamate to form glutamine, thus detoxifying the ammonia in the process. Glutamine is exported out of the glial cells into the extracellular fluid by SN/SA transporters in the SLC38 class (also known as SNATs) of solute transporters [53]. The free extracellular glutamine can then be taken back into neurons. Within neurons, phosphate-active glutaminase (GLS or PAG) recycles the glutamine to glutamate once again. Much of the glutamate within the brain is recycled through this glutamine cycle. Glutamate can be further processed into GABA via glutamate decarboxylase in GABAergic neurons.

Table 1 outlines human genes important for glutamatergic synthesis, metabolism, and signaling and known zebrafish paralogs. In most cases, zebrafish have multiple paralogs for each human gene; however, some genes in zebrafish only have a single ortholog. For example, zebrafish express *got1* (glutamic-oxaloacetic transaminase 1), the ortholog of *GOT1*, while there are two paralogs each of the vesicular glutamate transporters *SLC17A7* and *SLC17A6* (*slc17a7a* and *slc17a7b*, and *slc17a6a* and *slc17a6b*, respectively), with only one ortholog of *SLC17A8* (*slc17a8*). Zebrafish have three paralogs of glutamate-ammonia ligase *GLUL* (*glula*, *glulb*, and *glulc*) and two paralogs of glutaminase *GLS* (*glsa* and *glsb*) [60]. Zebrafish have 8 paralogous genes that code for AMPA type receptor subunits, 6 genes that code for KA type subunits, and 13 putative genes that code for NMDA type ionotropic receptors [61,62,63]. Humans have 8 subtypes of metabotropic receptors divided into three groups while zebrafish have 12 receptor subtypes similarly divided [64,65]. Currently there are 13 members of the *slc1a* family of EAATs transporters, with *slc1a8a*, *slc1a8b*, and *slc1a9* lacking corresponding paralogs in mammals due to a gene loss event by therian mammals [66,67].

During embryonic and post embryonic neurogenesis, proneural and neuronal cells express genes coding for basic helix-loop-helix (Bhlh) transcription factors and the patterns of expression can help identify neuronal populations [68]. The expression of neurogenin 1 (Neurog1)/Neurogenic differentiation 1 (Neurod; NeuroD1) has been linked to the development of glutamatergic neurons [69].

Identification of glutamatergic neurons in adult brains is often made based on the presence of transporter proteins. The SLC17A (VGLUT) genes *SLC17A6* and *SLC17A7* are often used as markers of glutamatergic neurons; however, glial cells can also express these transporters [33,70,71]. Glutamate itself is a poor marker due to its role in many metabolic pathways. Although the various receptor subunits are differentially expressed throughout the brain, glutamate receptors are also expressed on glia [72]. For a further account on distribution of these receptors in zebrafish please see Haug et al. [64] and Huang et al. [65].

### 3.2. GABA

GABA is the major inhibitory neurotransmitter in the central nervous system (CNS) and GABAergic neurons are widely present throughout the brain. As an inhibitory neurotransmitter, GABA mainly acts to modulate neural systems and the activity of postsynaptic cells [73]. GABA has been associated with the regulation of neural transmission and perturbances in the GABAergic system have been associated with epilepsy, depression, schizophrenia, and sleep dysfunction [74,75,76].

As reviewed by Ben-Ari and Reynolds et al. [77,78], in early development neurons have higher intracellular levels of chloride than mature neurons due to the expression of the sodium-potassium-chloride cotransporter 1 (NKCC1; SLC12A2) in the absence of potassium-chloride cotransporter 2 (KCC2; SLC12A5) expression [79]. Therefore, the classic inhibitory neurotransmitters GABA and glycine act to depolarize, and excite the immature neuron. Concurrent with neuronal maturation, SLC12A5 is expressed and this transporter reverses the chloride gradient, establishing the adult chloride gradient and causing GABA and glycine to act as inhibitory neurotransmitters [80].

GABA is synthesized in neurons through the GABA shunt. In the first step, α-ketoglutarate from the TCA cycle is transaminated by the 4-aminobutyrate transaminase (ABAT; GABA α-ketoglutarate transaminase; GABA-T) enzyme into l-glutamic acid. In the second step, glutamate decarboxylase (GAD) removes the carboxyl group and produces GABA. In mammals, two genes code for GAD, *GAD1* and *GAD2* (also known as *GAD*_67_ and *GAD*_65_, respectively) [81].

GABA is packaged into synaptic vesicles by the SLC32A1 solute carrier, also known as Vesicular GABA transporter (VGAT). Upon presynaptic depolarization, the vesicles are released into the synaptic space. SLC6A family members, SLC6A1, SLC6A11, and SLC6A12, also known as GABA transporters (GAT1, GAT3, and BGT1) are responsible for transporting GABA out of the synapse [82]. SLC6A13 (GAT2) does not appear to have a significant role in the brain [73]. Neurons may recycle the collected GABA back into synaptic vesicles or GABA can be metabolized to succinic semialdehyde by ABAT [83]. In glia, which lack GAD, the succinic semialdehyde is oxidized by succinic semialdehyde dehydrogenase (SSADH; aldehyde dehydrogenase 5 family member A1; ALDH5A) into succinic acid, which enters the TCA cycle and can be cycled through to α-ketoglutarate to again produce glutamine [73,84].

GABA has two classes of receptors: GABA_A_ and GABA_B_. As mentioned previously, GABA_A_ receptors are ligand-gated ion channel receptors and mediate postsynaptic membrane hyperpolarization through the influx of chloride through their integral channel [85]. Like all ligand-gated ion channels, GABA_A_ receptors are pentamers and 19 subunits in 7 classes provide basis of the regional variations and differential actions [86].

GABA_B_ receptors are metabotropic and therefore can mediate a variety of effects through their coupling with G proteins [87,88,89]. GABA_B_ receptors can activate certain potassium channels, regulate IP3, or inhibit cAMP production [89]. Presynaptic GABA receptors may inhibit presynaptic neurotransmitter release [90]. Two GABA_B_ receptor subunits have been identified in mammals, GABABR1 and GABABR2 (reviewed in [87,91]).

Table 2 list genes important for the synthesis, metabolism, and action of GABAergic neurons in humans and the known zebrafish paralogs. Zebrafish have a single copy of some GABAergic genes, such as the transaminase *abat*, the VGAT transporter *slc32a1*, and *aldh5a1 (SSADH).* Other genes such as *gad1* (glutamate decarboxylase), *slc6a1 (GAT1)*, and *slc6a11 (GAT3)* have two zebrafish paralogs [92]. Corresponding zebrafish orthologs or paralogs have not been identified for all human GABA receptor subunits (e.g., *GABRA2* and *GABRA4*), but some human genes have more than one paralog in zebrafish (e.g., *GABBR1*: *gabbr1a* and *gabbr1b*).

GAD has only been identified in GABAergic neurons, therefore providing a specific marker for GABA producing neurons. Additionally, zebrafish achaete-scute homolog 1a (Zash1a) expression has been linked to the development of GABA producing, inhibitory neurons, and has been used as a marker for GABAergic neurons during development [93,94].

In zebrafish, GABA is widely produced in the brain and spinal cord by interneurons [95]. The postembryonic (3 days post fertilization; dpf) subpallium, preoptic region, ventral and sections of the dorsal thalamus, and hypothalamus have been shown to produce GABA [93]. In the adult, GABA has been identified in the olfactory bulb, subpallium, preoptic, pretectal, ventral thalamic, hypothalamic, and posterior tubercular nuclei with scattered Gad1 positive cells in the pallial zones and the bed nucleus of the stria medullaris [96,97]. GABA, Gad2, Gabra1, and Gabbr1 are expressed in the zebrafish cerebellum and have a similar distribution to mammals [98].

### 3.3. Catecholamines

Dopamine, norepinephrine (noradrenaline), and epinephrine (adrenaline) are the major catecholamine neurotransmitters. Structurally, this group is characterized by a catechol group (benzene group with two adjacent hydroxyl groups), with an ethylamine side chain and an amine group. Catecholamines are considered modulatory neurotransmitters and have been linked to reward, movement, memory, and neuropsychiatric disorders [99,100,101].

Catecholamines are formed from the amino acid tyrosine and oxygen. Tyrosine hydroxylase (tyrosine 3-monooxygenase; TH) is the first enzyme in the synthesis pathway and is the rate limiting step. TH produces 3,4-dihydroxyl-l-phenylalanine (l-DOPA) with (6R)-l-erythro-tetrahydrobiopterin (BH4) and Fe^2+^ acting as cofactors in the hydroxylase step [102]. l-DOPA is converted to dopamine (DA) by the enzyme aromatic amino acid decarboxylase (AAAD), also known as DOPA decarboxylase [103]. AAAD is the preferred enzyme name as AAAD is also important in the monoamine serotonin synthesis pathway [82]. Although TH immunoreactivity is considered specific for dopaminergic neurons, AAAD can be found in non-monoamine producing neurons and glial cells. AAAD can also alternatively produce trace amines such as tryptamine, tyramine, and 2-phenylethylamine [104].

In noradrenergic and adrenergic neurons DA is converted to norepinephrine (NE) by dopamine-β-hydroxylase (dopamine β-monooxygenase; DBH) and NE can be further modified by phenylethanolamine-*N*-methyltransferase (PNMT) to epinephrine (EP).

Catecholamines are transported into vesicles through members of the SLC18 family, namely SLC18A2 (also known as VMAT2) in the brain (as reviewed by [105,106]). SLC18A2 can transport serotonin and histamine as well as catecholamines. Dopamine and norepinephrine transporters belong to the SLC6 family of carriers, with, at least in mammals, SLC6A2 functioning as the norepinephrine transporter (NET), and SLC6A3 as the dopamine transporter (DAT) [107].

Catecholamines are metabolized by a monoamine oxidase (MAO) enzyme. In humans, there are two isoforms of MAO in mammals, MAO-A and MAO-B, with different substrate specificity, pharmacology, and anatomic localization [108]. The catecholamine substrates are degraded into aldehydes, and aldehyde dehydrogenase and aldehyde reductase further degrade the products into alcohols or glycols respectively. Catechol-*O*-methyltransferase (COMT) adds a methyl group to catecholamines and their metabolites, which assists in elimination of the neurotransmitters and their metabolites. A major DA metabolite is 3,4-dihydroxyphenylacetic acid (DOPAC), which is the product of MAO and aldehyde dehydrogenase. DOPAC can be further metabolized by COMT to form homovanillic acid (HVA). Norepinephrine is mostly metabolized by MAO and aldehyde reductase, forming 3,4-dihydroxyphenylglycol (DHPG). Likewise, DHPG can be further metabolized to 3-methoxy-4-hydroxyphenylgylcol (MHPG) [104].

Catecholamines bind to G-protein-coupled receptors to modulate neurotransmission. As reviewed by Callier et al., there are five dopaminergic receptors in mammals: D1–D5, with D1 and D5 (D1-like) and D2–D4 (D2-like) sharing similar mechanisms [109]. D1-like receptors classically activate G_s_ G-proteins causing an increase in cAMP while D2-like activate Gα_i_/G_o_ G-proteins which inhibits adenylyl cyclase activity [110]. Beaulieu et al. extensively reviewed the mechanisms of dopamine receptor signaling [111]. Norepinephrine has nine receptors organized into three families, α_1_, α_2_, and β, each containing three receptors. α_1_ receptors activate G_q_/G_11_ proteins and activate phospholipase C and increase intra cellular calcium and protein kinase C activation. The α_2_ adrenergic receptors are coupled to G_i_/G_o_ proteins and inhibit adenylyl cyclase and stimulate phospholipase A2. The β adrenergic receptors are coupled to G_s_ and activate adenylyl cyclase [104].

Originally two Th encoding paralogs were identified in teleosts, *th1* and *th2* [112]. *th1*-negative, *th2*-positive neurons were identified in zebrafish brain [113,114,115] and although these neurons appeared to be immunoreactive for *slc18a2 (vmat2)*, *aaad*, and *slc6a3 (dat)*, consistent with a dopaminergic phenotype, it was discovered that the gene encoded by *th2* appears to function as a tryptophan hydroxylase when isolated in vitro [115,116]. Therefore, *th1* is referred to as *th* by the Zebrafish Information Network (ZFIN) and is considered the only tyrosine hydroxylase in zebrafish. Currently, only one ortholog of *AAAD (aaad)*, *DBH* (*dbh*), and of the transporters *SLC18A2* (*slc18a2*), *SLC6A2* (*slc6a2*), and *SLC6A3* (*slc6a3*) have been identified. Although mammals have two isozymes, MAO-A and MAO-B, zebrafish have only one paralogous enzyme, Mao (also known as Zmao) [117]. Zebrafish Mao may have a structure and function more similar to MAO-A, but is inhibited by *deprenyl*, a MAO-B specific inhibitor [118,119]. Two putative *COMT* genes have been identified, *comta* and *comtb*. However, the gene products have not been fully characterized, and some protein products may not be functional [16,120]. Table 3 outlines known paralogs of dopamine and adrenergic receptors [121,122]. The receptor genes *drd1a* and *drd1b* likely have D1-like activity and the other receptor genes likely have D2-like functions [122].

**Figure 1:**
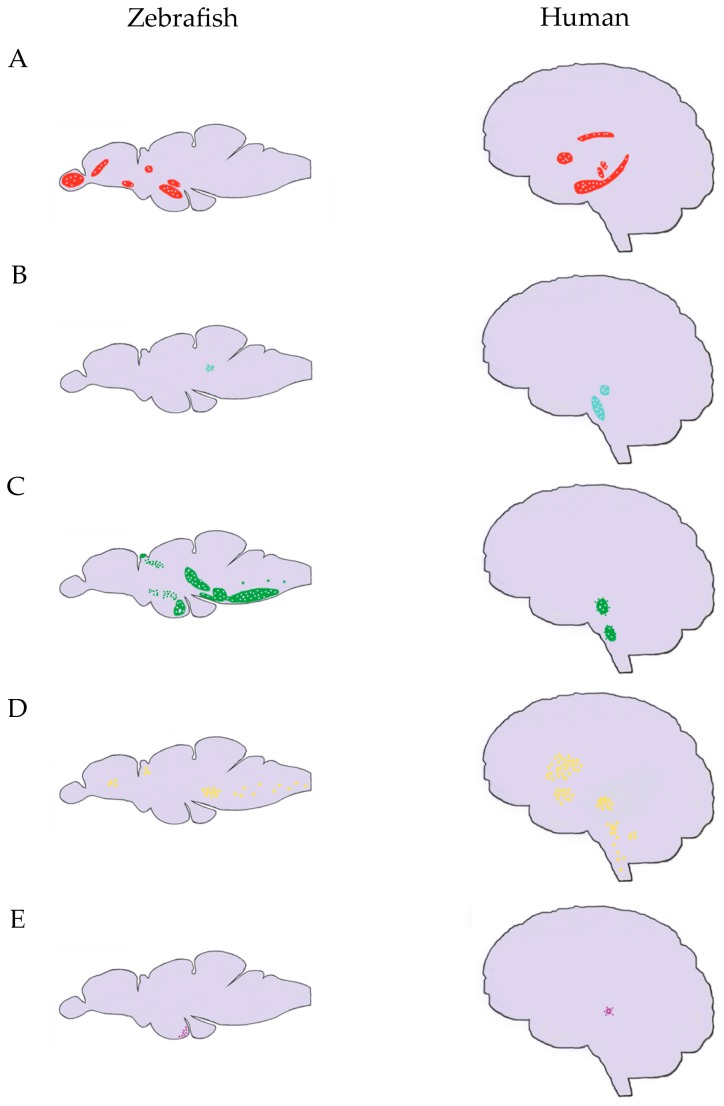
Distribution of Modulatory Neurotransmitters in the Zebrafish and Human Brain. (**A**) Distribution of DA in the zebrafish (left) and human (right) brains (red); (**B**) Distribution of NE in zebrafish (left) and human (right) brains (blue); (**C**) Distribution of 5-HT in zebrafish (left) and human (right) brains (green); (**D**) Distribution of ACh in zebrafish (left) and human (right) brains (yellow); (**E**) Distribution of histamine in zebrafish (left) and human (right) brains (purple). Brains are not to scale with some simplification of systems that are represented. DA, dopamine; NE, norepinephrine; 5-HT, serotonin; ACh, acetylcholine. Neurotransmitter distributions synthesized from [16,127,128,129,130,131,132,133].

In order to help compare neuroanatomy between species, dopaminergic populations have been labeled numerically based on rostral to caudal location in the brain. As reviewed by Schweitzer et al. [123], Rink and Wullimann [124] labeled the dopaminergic populations 1–8 in larvae and 0–8 in adults, with 0 representing the ventral thalamic area. Sallinen [119] used a 17 population classification scheme. Both classification systems localize dopaminergic neurons to the olfactory bulb, subpallium, posterior tuberculum, hypothalamus, and pretectum [123]. Zebrafish brain lack a substantia nigra and ventral tegmental area, however, the posterior tuberculum has populations of dopaminergic cells with projections that extend to the subpallium and spinal cord [125,126]. Figure 1 outlines the locations of the modulatory neurotransmitters in humans and zebrafish.

Neurons producing dopamine or norepinephrine also produce either glutamate or GABA and therefore have two transmitter profiles. Dopaminergic neurons in the dopaminergic posterior tubercular groups 2, 4, and 6 and hypothalamic group 5 and some norepinephrine producing cells in the area postrema produce glutamate and all other dopamine or norepinephrine producing neurons produce GABA [134].

DBH, the enzyme that converts dopamine to epinephrine, is also present and is used as a marker of adrenergic neurons. Dbh is only found in zebrafish hindbrain, specifically at the locus coeruleus [135,136]. The locus coeruleus projects to the pallium, the subpallium and the thalamus [137,138].

### 3.4. Serotonin

The neurotransmitter serotonin (5-hydroxytrptamine; 5-HT) is a biologic amine. 5-HT has an indole nucleus with a hydroxyl group and an amine group. l-tryptophan is the base of the molecule, with dietary protein being the major source. 5-HT is a modulatory neurotransmitter and has been associated with brain development, appetite, motor function, arousal and mood, neuroendocrine function, circadian rhythms, and depression [139,140,141,142].

The first enzyme in the 5-HT synthesis pathway is tryptophan hydroxylase (l-tryptophan-5-monooxygenase; TPH), which converts tryptophan to 5-hydroxytryptophan (5-HTP). The conversion of tryptophan to 5-HTP is considered the rate limiting step in the synthesis pathway and is unique to serotonergic neurons. In mammals there are two genes that code for TPH. *TPH1* is expressed in the periphery and *TPH2* is expressed exclusively in the brain and can be used as a marker for serotonergic neurons [143]. Once formed, 5-HTP is quickly converted to 5-HT by AAAD.

5-HT is transported into synaptic vesicles by vesicular transporter SLC18A2 (VMAT2) and released from the vesicle via exocytosis [144]. The serotonin transporter SLCl6A4 (also known as SERT) is responsible for the uptake/reuptake of 5-HT, although glia and non-serotonergic neurons can take up serotonin through organic cation transporter (OCT), plasma membrane monoamine transporter (PMAT), or through SLC6A2 or SLC6A3 (NET and DAT, respectively) [145].

5-HT is metabolized by MAO to 5-hydroxy-indolecetaldehyde which is rapidly metabolized by an aldehyde dehydrogenase to form 5-hydroxyindoleacetic acid (5-HIAA), the major metabolite of 5-HT [145].

In mammals there are three families of 5-HT receptors (HTR) that act through G-proteins, the 5-HT_1_ family, the 5-HT_2_ family, and a family that includes the 5-HT_4_, 5-HT_6_, and 5-HT_7_ receptors [142]. 5-HT_3_ is a cation specific ligand-gated ion channel and is considered its own family [145]. The 5-HTR_1_ family generally acts to inhibit adenylyl cyclase through the G_i/o_ family of G proteins while the 5-HTR_2_ family acts through G_q/11_ family G proteins by stimulating phospholipase C. The 5-HTR_4_, 5-HTR_6_, and 5-HTR_7_ family mainly act through the G_s_ family G proteins to stimulate adenylyl cyclase [146].

As a result of the teleost gene duplication event, zebrafish have four paralogous genes encoding TPH, although the nomenclature has not been standardized. Bellipanni et al. first identified two paralogs of *TPH* in the developing zebrafish brain, *tphD1*, expressed in the preoptic nuclei and the posterior tubercular in the diencephalon, and *tphD2*, expressed in the pineal gland and transiently in the preoptic nuclei [147]. Teraoka et al. identified a third paralog of Tph, *tphR*, expressed in the raphe nuclei and pineal gland [148]. In later reviews of the serotonin neurotransmitter system, these genes are referred to as *tph1a*, *tph1b*, and *tph2*, respectively [149,150,151]. Further research found that zebrafish *th2* encodes for a fourth *tph* gene in the ventral diencephalon and caudal hypothalamus [116]. This gene has also been called *tph3* to better reflect its function [150]. As mentioned previously, zebrafish have only one ortholog of *AAAD* and *MAO* [117]. There are two paralogs of the serotonin transporter gene *SLC6A4*, *slc6a4a* and *slc6a4b* [152]. In mammals, over 15 *HTR* genes have been identified. Table 4 outlines the critical genes in serotonin synthesis and metabolism as well as listing known paralogous genes for the serotonin HTR receptor families [121].

As reviewed by Lillesaar [149], in larval zebrafish 5-HT positive cells are located in the pineal gland, the pretectum, the posterior tuberculum, the hypothalamus, and the superior and inferior raphe. Panula et al. outlined a labeling scheme for the adult zebrafish with serotoninergic nuclei identified in the pretectal complex, the anterior, intermediate, and posterior paraventricular organ nuclei, the dorsal, median, and ventrolateral raphe, the inferior raphe, and the caudal raphe [16,150]. Adult zebrafish also have scattered serotonergic neurons within the medulla oblongata [137]. The distribution of 5-HT in the zebrafish brain compared to humans is shown in Figure 1C.

When identifying serotonergic populations, the Ets-domain transcription factor pet1 (pheochromocytoma 12 ETS [E26 transformation-specific]) is a specific developmental marker of the raphe serotonergic nuclei [151]. TPH is typically used as the serotonergic specific marker in imaging studies [149].

### 3.5. Acetylcholine

Acetylcholine (ACh) is the major neurotransmitter in the parasympathetic nervous system and is the neurotransmitter at neuromuscular junctions [153]. Additionally, ACh neurotransmission is widespread in the CNS and can help modulate the release of other neurotransmitters such as GABA, and has been implicated in arousal, reward, and learning and memory [153,154,155].

ACh is formed from acetyl-CoA and choline via choline acetyltransferase (ChAT). Two SLC family transport systems, a high affinity (SLC5A7; HAChU) and a low affinity (SLC44 family; LAChU), concentrate choline in terminals to provide a reserve for ACh synthesis. SLC44 is present ubiquitously throughout the body, but SLC5A7 is only found in cholinergic nerve termini [156]. The rate of SLC5A7 transport is regulated by the rate of ACh release and SLC5A7 is the rate limiting step in ACh production. Once ACh has been synthesized by ChAT it is packaged into vesicles by vesicular ACh transporter (VAChT) which is coded by SLC18A3 [106]. ACh is then released in quanta into the synaptic cleft in a calcium dependent manner. Unlike other neurotransmitters which have transporter mediated uptake/reuptake to clear the synapse, ACh is metabolized by acetylcholinesterase (AChE) within the synaptic space and broken into acetate and choline [156].

There are two major types of cholinergic receptors, nicotinic (nAChR) and muscarinic receptors (mAChR). The receptors were respectively named after nicotine and muscarine, their drug agonists. nAChRs are a member of the cys-loop family of ligand-gated ion channel receptors and are formed by combinations of receptor subunits [156,157]. In mammals, there are 17 nicotinic receptor subunits including muscle and neural specific subunits. nAChRs are located throughout the brain, though many subtypes of nAChR are located on presynaptic termini or cell bodies and function to modulate neurotransmitter release [154]. mAChRs are G-protein-coupled receptors and are located throughout the CNS and PNS. There are five subtypes of mAChR receptors in mammals, M1–M5. M1, M3, and M5 couple to G_q/11_ family proteins to increase phospholipase C. M2 and M4 receptors act to decrease adenylyl cyclase through G_i_/G_o_ receptor activation. mAChR are expressed throughout the brain, but are not uniformly distributed (reviewed by Brown [158]).

The important genes in the cholinergic system are outlined in Table 5. Zebrafish have two paralogs of the HAChU *SLC5A7 (slc5a7a* and *slc5a7b*), although *slc5a7b* has not been well described. The gene that produces ChAT has two paralogs in zebrafish, *chata* and *chatb*. *slc18a3a* and *slc18a3b* are two paralogs of *SLC18A3* (VAChT). There is only one paralog of *ACHE* (*ache*). Currently, there are 12 putative genes encoding nAChR subunits in zebrafish and 10 putative paralogs of mAChR [159,160,161].

Cholinergic neurons have been identified through immunohistochemical staining against choline-acetyltransferase (ChAT) [94,162]. In zebrafish, cholinergic neurons are found in both the brain and spinal cord, specifically in the octavolateralis cells and modulatory or sensory neurons, the ventral telencephalic area, the central, dorsal, and subcommissural nuclei of the ventral telencephalic areas, the preoptic area, dorsal thalamus, pretectal nuclei, hypothalamus, optic tectum, and tegmentum [137,163,164,165]. The distribution of ACh in zebrafish and human brains is shown in Figure 1D.

### 3.6. Histamine

Histamine is a signaling molecule present in many tissues, serving functions in the stomach, skin, and immune systems. Histamine also has a role in neurotransmission [166]. Within the CNS, histamine is associated with wakefulness, feeding and drinking, and learning and memory [130,167,168].

The structure of histamine, 2-(4-imidazolyl)ethylamine, is similar to 5-HT, NE, and EP, but histamine has an imidazole nucleus and therefore has tautomeric properties that may be associated with receptor affinity. In mammals, mast cells of bone marrow origin reside in perivascular spaces, choroid plexus, and meninges and can produce significant amounts of histamine within the brain although the only neurons that produce histamine are located within hypothalamic tuberomamilary neurons within the posterior hypothalamus [169]. Zebrafish, on the other hand, do not have stores of histamine outside of the brain, suggesting any histamine is of importance to neurotransmission [130,170]. The periventricular cells of the caudal hypothalamus are the only cell group that contains histaminergic neurons in zebrafish brain, similar to mammals, although the axons project throughout the CNS [137,170]. These histamine producing neurons also contain other signaling molecules including GABA, neuropeptides, and thyrotropin-releasing hormone [171].

Histamine is synthesized by l-histidine decarboxylase (HDC). The rate of biosynthesis is controlled by the availability of l-histidine and the rate limiting enzyme, HDC. Once formed, histamine is transported into vesicles by SLC18A2 (VMAT2) [172]. Most histamine in the brain is released via non-synaptic mechanisms and often acts on both presynaptic and postsynaptic receptors. There is no evidence of a neuronal histamine transporter [169].

The metabolism of histamine can occur either by diamine oxidase (DAO; amine oxidase AOC1), which oxidizes histamine to imidazole acetic acid (IAA), or by histamine *N*-methyltransferase (HNMT), which methylates histamine and forms tele-methylhistamine (t-MH) and is then further metabolized by MAO (MAO-B in mammals) to tele-methylimidazole acetic acid (t-MIAA). The methylation metabolism pathway is more common in vertebrate brains and HMT is widely distributed throughout the brain [168,169].

Mammals have four histamine receptors that are found in the brain, H1, H2, H3, and H4. H1 and H2 are considered excitatory while H3 is inhibitory and often acts as an autoreceptor. H4 is the most recently discovered but does appear to localize to the brain [173]. Histamine receptors are linked to G proteins in both neurons and glia. H1 receptors are linked to G_q_ and stimulate phospholipase C. H2 receptors may couple with G_q_ or G_s_ but act through the stimulation of adenylyl cyclase. H3 receptors are linked to G_i/o_ and inhibit adenylyl cyclase. H3 receptors may also activate MAPK, Akt/GSK-3β, and phospholipase A_2_ pathways [174,175,176]. The H4 receptor is similar to the H3 receptor and acts through G_i/o_ proteins to inhibit adenylyl cyclase [173].

In zebrafish there is one ortholog each of *HDC* (l-histidine decarboxylase), *AOC1* (diamine oxidase), and *HNMT* (histamine *N*-methyltransferase) (Table 6). To date, there are four known genes in zebrafish that code for histamine receptors, *hrh1*, *hrh2a*, *hrh2b*, and *hrh3* [177]. In zebrafish, histamine containing neurons have been localized only to the ventrocaudal hypothalamus, though the projections are widespread [137,178]. The distribution of histamine in the zebrafish brain compared to humans is shown in Figure 1E.

### 3.7. Glycine

Glycine is an amino acid that serves as a signaling molecule and neurotransmitter in the brainstem and spinal cord. Glycine is the simplest amino acid, with only a hydrogen for its side chain. Glycine, typically considered an inhibitory neurotransmitter, is involved with interneuron differentiation in neurodevelopment, mediation of spinal reflexes, and reflex behaviors such as breathing [179,180].

Glycine can be formed through the conversion of serine to glycine with either glycine dehydrogenase (GLDC), also known as glycine decarboxylase (GDC) or glycine-cleavage system, or serine hydroxylmethyltransferase (SHMT). Glycine is transported into synaptic vesicles via the vesicular inhibitory amino acid transporter, SLC32A1 (VIAAT, also known as vesicular GABA transporter (VGAT)) [180].

Glycine is transported out of the synaptic cleft by glycine transporters. Two glycine transporters, SLC6A9 (GLYT1) and SLC6A5 (GLYT2), have been identified in mammals [180]. SLC6A9 and SLC6A5 transporters are expressed on both astrocytes and postsynaptic neurons [181].

The glycine receptors are in the ligand gated ion channel superfamily. In mammals, glycine receptor subunits arise from two separate gene families. The α gene family has four subunit genes (α_1_–α_4_) and the β gene group has only one member (reviewed by Bowery and Smart [182]). Glycine also acts on NMDA receptors and modulates the amplitude and time course of the glutamate-elicited response [183]. Interestingly, although no metabotropic counterparts have been identified, the glycine receptor subunit α can interact with G protein βγ subunits, which potentiates the response to glycine [184].

As outlined in Table 7, zebrafish tend to have a single known ortholog for most of the genes important for glycine metabolism [185,186]. The exception is the presence of two paralogs for glycine receptor α subunit 4 gene and the glycine receptor β gene. Glycinergic neurons are identified via positive immunoreactivity for glycine transporter (*slc6A9*) immunohistochemistry. In the developing zebrafish, glycine producing cells are limited to the hindbrain and spinal cord, and appear as early as 20 h post fertilization (hpf) [187,188]. In adult zebrafish, the vast majority of glycinergic neurons are within the medulla oblongata, though a few positive neurons are within a ventral tegmental equivalent nucleus [129,187].

### 3.8. Other Neurotransmitters

Other substances can also act as neurotransmitters in the brain, including purines, peptides, nitric oxide, and endocannabinoids [189,190]. Please see Rico et al. [11] for a review of the purine nucleotides and nucleosides in zebrafish and Panula et al. [16] for a review of neuropeptides.

## 4. Evaluation of Neurotoxicity

The neurotransmitter systems can serve as targets of chemical toxicity. The individual enzymes, transporters, and receptors may be altered by chemical toxicants through changes in gene expression or changes in activity of the enzyme or receptor. Multiple methods can be used to evaluate neurotransmitter systems in zebrafish, from the measuring of neurotransmitter levels in the brain, to evaluation of gene expression, to the functional testing of behavioral assays. These methods are reviewed below.

### 4.1. Evaluation of Neurotransmitters

In the evaluation of chemical toxicants, measuring neurotransmitter levels can provide information on the functional alterations in the brain resulting from chemical treatment. The quantity of neurotransmitters can be measured in the zebrafish brain. Sallinen et al. [119] and Chattererjee et al. [191] have both described methods for measuring neurotransmitter levels via high performance liquid chromatography (HPLC) that have been modified and used in other laboratories [192,193].

Experimental neurotransmitter results may be comparable within one laboratory, but not comparable across multiple laboratories due to differences in experimental methods and equipment used. Furthermore, one difficulty in comparing neurotransmitter levels across studies and laboratories is the lack of reference intervals and the tendency to normalize neurotransmitter values to controls rather than reporting absolute values, which limits comparison. For example, Pan et al. [194] reported the difference in neurotransmitter levels between the AB and short-fin wildtype strains of zebrafish as a ratio between zebrafish strains, rather than average concentration. Table 8 lists reported neurotransmitter levels from control zebrafish. Comparison of values in the table is difficult between studies in part to different methodologies used for normalization. Neurotransmitter levels can be normalized to protein content or number of fish pooled for the sample, and it is difficult to equate between the two methods.

Other methods for the measurement of neurotransmitters include an analytical method described by Tufi et al. [195] of hydrophilic interaction liquid chromatography (HILIC) coupled to tandem mass spectrometry (MS/MS) that has been used to measure neurotransmitter levels in zebrafish larvae. The levels of neurotransmitters and major metabolites were measured in 0–6 dpf zebrafish larvae. Additionally, Jones et al. [196] describe a technique to detect neurotransmitter release and reuptake in brain tissue slices through fast scan cyclic voltammetry (FSCV).

### 4.2. Evaluation of Gene Expression Changes in Neurotransmitter Pathways

Chemical toxicants can affect the expression of genes, and evaluation of these changes can provide information on pathways that may be altered due to chemical exposure. Quantitative PCR (qPCR) can be used to evaluate alterations in gene expression [198]; however, the information is limited to the number of genes investigated.

Microarray studies have been used to evaluate gene expression after chemical exposure in zebrafish [199,200,201,202]. The value of transcriptomic evaluations in zebrafish toxicology studies is well recognized, especially for environmental toxicology [12,203]. Next generation technologies such as RNA-seq are providing precise and powerful options for evaluating the transcriptome [203,204]. Please see the review by Wang et al. [204] and Aanes et al. [205] for information on RNA-seq and its application in zebrafish.

### 4.3. Evaluation of Neurobehavior

The significance of apparent alterations in gene expression and changes in neurotransmitter levels is uncertain without having differences in phenotype. Zebrafish are an accepted model of neurobehavior [206,207,208,209,210,211,212,213,214,215] and have been used to study stress, anxiety-like behavior, and depression, with decreased movement typically associated with anxiety [216,217,218]. Zebrafish are increasingly being used in neurobehavioral research to evaluate learning and neuropsychiatric disorders (reviewed by [207,208,212,219]). Neurobehavioral tests have been developed for both larval and adult zebrafish to assess anxiety-like behavior. Changes in neurobehavior can substantiate changes observed through neurotransmitter analysis or through transcriptomic evaluation; however, changes in neurobehavior cannot be localized to a specific neurotransmitter, pathway, or protein. Please refer to Kalueff et al. [219] and Parker et al. [220] for recent reviews of neurobehavior in zebrafish toxicology research.

### 4.4. Visualization of Neurotransmitters and Neurotransmitter Systems

The visualization of neurotransmitters has been important for evaluating the location, relative quantity, and pathologic alterations of neurotransmitters and neurotransmitter pathway components. A multitude of techniques, including immunohistochemistry, in situ hybridization, immunofluorescence, calcium indicators, selective fluorescent reporters [221,222,223,224,225,226] (and reviewed by [227]) have been used to study neuroanatomy and neurotransmitter systems in zebrafish. The imaging of neurotransmitters, transporters, and receptors in zebrafish brains has classically been used in the study of developmental biology [68] and recently for neural systems mapping [226,228]. A recent review by Arrenberg and Driever [228] highlights the use of optogenetics and calcium indicator activity probes in the development of functional maps of the zebrafish brain. Currently this methodology has been underused in studies of chemical toxicity, but could provide powerful information when combined with the other approaches mentioned.

### 4.5. Pharmacology Screens

The advantages of the zebrafish model system allow for high throughput screening of pharmacologic agents which can help identify neuropathways, mechanisms of toxicity, possible therapeutic drugs, and help classify zebrafish behavior (reviewed by [229,230,231,232,233,234]). The neuropharmacology of the monoamine neurotransmitters was reviewed by Maximino and Herculano [235]. Kalueff et al. [219] have also recently written a highly recommended review that evaluates the zebrafish system in neurobehavior and pharmacology screenings.

## 5. Specific Examples of Chemical Toxicity Targeting Neurotransmitter Systems

Zebrafish have been used as a model organism to study the effect of multiple classes of chemicals on neurodevelopment and neural function. Classes of chemicals examined include drugs, especially ethanol, pesticides, and metals. The significant methods and findings from the literature are summarized below and in Table 8.

### 5.1. Drugs

The effects of alcohol have been extensively studied in zebrafish. Rico et al. [236] found that adult zebrafish exposed to ethanol had increased AChE activity in a 1% ethanol treatment group although the mRNA levels of AChE were decreased, suggesting post-transcriptional or post-translational modifications to AChE. Chatterjee and Gerlai [191] found that adult zebrafish exposed to ethanol for 1 h had an increase in brain dopamine levels at all treatment levels, while 5-HT and 5-HIAA increased at the highest (1%) ethanol group. Chatterjee and Shams [237] found a similar increase in dopamine and 5-HT after acute ethanol exposure in AB strain zebrafish, but not in SF strain zebrafish. Puttonen et al. [238], found that larval Turku strain zebrafish with acute exposure to ethanol had increased locomotor activity at lower treatment levels, decreased locomotor activity at the highest treatment level (3%), upregulation of *hdc* (histidine decarboxylase), *th1*, and *th2* at higher treatment levels, no alterations in the dopaminergic and histaminergic systems according to in situ hybridization and immunohistochemistry, and a decrease in dopamine levels, as measured by HPLC. These results suggest that ethanol has the ability to alter the cholinergic, dopaminergic, and histaminergic neurotransmitter systems and that neurobehavior can be a sensitive measure of altered neurotransmitter systems.

The effects of alcohol on zebrafish behavior appear to have an inverted U shaped dose-response, with increased locomotor activity and shoaling behavior at lower doses and decreased locomotor activity and shoaling behavior at higher doses, as well as either increased or decreased measures of anxiety [237,239,240,241,242]. Although this finding appears to be dependent on the specific zebrafish strain (AB, SF, WIK, or Turku strain) used and therefore, needs further analysis [237,238,239,243]. Bailey et al. found juvenile AB strain zebrafish with developmental exposure to ethanol had increased locomotor activity overall and after stress or anxiety inducing stimuli [244]. A latent learning neurobehavioral assay performed by Luchiari et al. suggested that AB strain zebrafish have impaired memory recall after ethanol exposure [245]. Echevarria et al. have reviewed additional behavioral outcomes of ethanol exposure in zebrafish [246].

Zenki et al. [247] found that alcohol and its metabolite acetaldehyde decreased the activity of glutamate transporters, based on measuring the in vitro rate of glutamate uptake in tissue sections, in adult zebrafish and that acetaldehyde was more toxic than ethanol when measuring cell viability via 3(4,5-dimethylthiazol-2-yl)-2,5-diphenyl tetrazolium bromide (MTT) reduction and extracellular lactate dehydrogenase (LDH) activity.

Nicotine is often used to study nicotinic cholinergic receptors, but nicotine also is a drug known for anxiolytic effects. Levin et al. [248] and Bencan and Levin [249], found that adult zebrafish exposed to nicotine had reduced time spent in the bottom of a novel tank (novel tank test) and found that the anxiolytic effect was mediated through the Chrna7 and Chrna4b receptor subunits [248,249]. Levin and Chen (2004), also found that adult zebrafish exposed to low dose nicotine had improved memory based on a 3-chambered tank test for learning assessment while zebrafish exposed to higher concentrations of nicotine had impaired memory function [250].

### 5.2. Pesticides

Pesticides represent a broad group of chemicals that include herbicides, insecticides, fungicides, and rodenticides. Some pesticides are important environmental toxicants while others are more acutely toxic to humans (for example, during application or manufacturing).

The effects of organophosphate pesticides on brain acetylcholinesterase are well known, however, exposure to organophosphates can also affect other brain neurotransmitter systems. Eddins et al. [251] studied the effects of developmental exposure of zebrafish to chlorpyrifos. Adult zebrafish previously exposed to chlorpyrifos had greater startle responses in a startle response and habituation behavioral assay that persisted into the habituation period. Additionally, decreased dopamine and serotonin levels and increased transmitter turnover were measured in larval zebrafish while only the decreased dopamine persisted to adulthood [251].

Atrazine is a commonly used herbicide in the Midwestern United States that often contaminates drinking water supplies. Wirbisky et al. [199] found decreased levels of the serotonin metabolite 5-HIAA and decreased serotonin turnover (5-HIAA/5-HT) in the brain of adult female zebrafish aged 9 months that were exposed to atrazine only during embryogenesis. Transcriptomic analysis via microarray identified multiple molecular pathways related to brain development, function, and behavior that were altered in the treatment groups including several targets associated with the serotoninergic system [199].

Semicarbazide is a contaminant formed from the breakdown of azodicarbonamide, a chemical used to treat flour. Semicarbazide derivatives have also been used as herbicides. Adult zebrafish exposed to semicarbazide for 96 h had increased expression of *gad1* while adult zebrafish with 28 day exposure had down regulation of *gad1*, *gabrr1*, and *gabbr2* [252]. Yu et al. [252] suggest the alterations of the GABAergic pathway genes could indirectly result in alterations in the hypothalamus-pituitary-gonadal axis.

Strychnine is commonly used as rodent bait. Roy et al. [253] found that zebrafish embryos treated with strychnine had decreased expression of *glra4* at 24 and 48 hpf, *gad1* at 24–96 hpf, and *slc17a6a* and *slc17a6b* (VGLUT2) at 48 hpf.

### 5.3. Metals

Some metals, such as lead and mercury are important environmental toxicants [254,255,256]. Developmental lead exposure is linked to a number of CNS effects, including lowered IQ and attention deficit disorders [257,258,259,260]. Wirbisky et al. [193] found that developmental exposure to lead resulted in altered expression of GABAergic pathway genes including *gad2*, *gad1b*, *slc6a1* (*gat1*), *slc32a1* (*vgat*), *gabbr1*, and *gabbr1a* as well as altering GABA levels during development. Furthermore, Lee and Freeman [261] found that adult zebrafish with a developmental lead exposure had altered gene expression in pathways associated with neurodevelopment and neurotransmission.

In addition, studies have also started to evaluate the impact of mercury exposure on neurotransmitter systems. The toxicity of mercury depends on its chemical form. Methylmercury is associated with Minamata disease and nervous system impairment. Cambier et al. [198] fed adult male fish food contaminated with methylmercury and found changes in gene expression in the GABA synthesis and metabolism pathways. Inorganic mercury is associated with acute toxicity and renal failure; however, Richetti et al. [262] found that adult zebrafish exposed to mercury chloride and lead acetate had decreased activity of acetylcholinesterase, but no alterations in the gene expression of AChE.

## 6. Conclusions

The neurotransmitter systems are highly conserved between zebrafish and mammals, making the zebrafish model a powerful tool for the study of mechanisms of chemical neurotoxicity. Although there are some differences in neurochemistry, the pathways of neurotransmitter synthesis, metabolism, and action are highly conserved across species. Although some genes have multiple paralogs in zebrafish, further research into these paralogs may help identify new functions of genes in humans. Furthermore, further research into neurotransmitter receptors may provide more information about basic neurological systems and connectivity.

The nervous system is an important target of chemical toxicants. Neurotransmitter levels, gene expression, and neurobehavior can be evaluated after chemical treatment to discover toxicant based changes in the nervous system. New technologies provide more sensitive and powerful methods; however, the integration of these methods along with other techniques, such as imaging and activity assays, will be necessary to determine the mechanisms and pathogenesis of chemical toxicant related alterations. Overall, relatively few studies have focused on alterations of neurochemical systems in zebrafish, but the completed studies support the utility and application of zebrafish in neurochemical toxicology.

## Figures and Tables

**Table 1:** Zebrafish genes involved in glutamate neurotransmission.

Common Name	Human	Zebrafish	Gene ID	RefSeq	ZFIN ID
Glutamic-oxaloacetic transaminase 1	*GOT1*	*got1*	406330	NM_213057	ZDB-GENE-040426-2003
Vesicular glutamate transporter 1 (VGLUT1)	*SLC17A7*	*slc17a7a*	795293	NM_001098755	ZDB-GENE-050105-5
		*slc17a7b*	100331980	XM_009297642	ZDB-GENE-131125-32
Vesicular glutamate transporter 2 (VGLUT2)	*SLC17A6*	*slc17a6a*	494492	NM_001009982	ZDB-GENE-050105-4
		*slc17a6b*	100149756	NM_001128821	ZDB-GENE-030616-554
Vesicular glutamate transporter 3 (VGLUT3)	*SLC17A8*	*slc17a8*	563467	NM_001082835	ZDB-GENE-060503-416
Glutamate-ammonia ligase (Glutamine synthetase)	*GLUL*	*glula*	100000775	NM_181559	ZDB-GENE-030131-688
		*glulb*	336473	NM_182866	ZDB-GENE-030131-8417
		*glulc*	566165	NM_001075114	ZDB-GENE-060929-540
Glutaminase	*GLS*	*glsa*	564147	NM_001045044	ZDB-GENE-050204-3
		*glsb*	564746	XM_688079	ZDB-GENE-030616-550
Ionotropic Reptors					
AMPA Receptors	*GRIA1*	*gria1a*	798689	NM_205598	ZDB-GENE-020125-1
		*gria1b*	403044	NM_205730	ZDB-GENE-020125-2
	*GRIA2*	*gria2a*	170450	NM_131894	ZDB-GENE-020125-3
		*gria2b*	170451	NM_131895	ZDB-GENE-020125-4
	*GRIA3*	*gria3a*	170452	NM_198339	ZDB-GENE-020125-5
		*gria3b*	368416	NM_198360	ZDB-GENE-030616-53
	*GRIA4*	*gria4a*	407735	NM_214806	ZDB-GENE-020125-7
		*gria4b*	336069	NM_212752	ZDB-GENE-030131-8013
Kainate Receptors	*GRIK1*	*grik1a*	798001	XM_009305317	ZDB-GENE-030131-6502
		*grik1b*	561540	XM_684948	ZDB-GENE-070821-1
	*GRIK2*	*grik2*	556013	XM_009300832	ZDB-GENE-080414-1
	*GRIK3*	*grik3*	100334689	XM_009300849.1	-
	*GRIK4*	*grik4*	556582	XM_009291736	ZDB-GENE-070821-5
	*GRIK5*	*grik5*	798791	NM_001328156	ZDB-GENE-070821-6
NMDA Receptors	*GRIN1*	*grin1a*	767745	NM_001076714	ZDB-GENE-051202-1
		*grin1b*	100005675	NM_001144131	ZDB-GENE-051202-2
	*GRIN2A*	*grin2aa*	563297	XM_686662	ZDB-GENE-070424-129
		*grin2ab*	570493	XM_009306215	ZDB-GENE-070424-223
	*GRIN2B*	*grin2ba*	-	-	ZDB-GENE-090821-2
		*grin2bb*	559976	NM_001128337	ZDB-GENE-061207-27
	*GRIN2C*	*grin2ca*	100003342	XM_002661129	ZDB-GENE-070822-3
		*grin2cb*	100333648	XM_009306796	ZDB-GENE-100308-2
	*GRIN2D*	*grin2da*	449864	XM_009294079	ZDB-GENE-041008-124
		*grin2db*	-	-	ZDB-GENE-100920-7
	*GRIN3A*	*grin3a*	564832	XM_009305086	ZDB-GENE-130530-780
		*grin3ba*	566411	XM_009298558	ZDB-GENE-070912-354
	*GRIN3B*	*grin3bb*	100333101	XM_009305920	ZDB-GENE-131122-77
Metabotropic Receptors					
Group I	*GRM1*	*grm1a*	555576	NM_001044788	ZDB-GENE-030131-7893
		*grm1b*	100150246	NM_001302252	ZDB-GENE-090821-3
	*GRM5*	*grm5a*	568406	NM_001328710	ZDB-GENE-090821-9
		*grm5b*	100332913	NM_001302238	ZDB-GENE-090821-6
Group II	*GRM2*	*grm2a*	336153	NM_001302225	ZDB-GENE-030131-8097
		*grm2b*	564461	NM_001287547	ZDB-GENE-060201-5
	*GRM3*	*grm3*	565256	NM_001128343	ZDB-GENE-061009-13
Group III	*GRM4*	*grm4*	567181	NM_001302241	ZDB-GENE-030131-5781
	*GRM6*	*grm6a*	568484	NM_001123292	ZDB-GENE-060208-1
		*grm6b*	565450	NM_001080020	ZDB-GENE-021120-2
	*GRM7*	*	-	-	-
	*GRM8*	*grm8a*	792371	NM_001302228	ZDB-GENE-110421-2
		*grm8b*	569768	NM_001287539	ZDB-GENE-110421-3
Glutamate Transporters	*SLC1A1*	*slc1a1*	436939	NM_001002666	ZDB-GENE-040718-414
	*SLC1A2*	*slc1a2a*	560802	NM_001190305	ZDB-GENE-100422-11
		*slc1a2b*	335836	NM_199979	ZDB-GENE-030131-7779
	*SLC1A3*	*slc1a3a*	323439	NM_212640	ZDB-GENE-030131-2159
		*slc1a3b*	556181	NM_001190303	ZDB-GENE-090708-3
	*SLC1A4*	*slc1a4*	368885	NM_001002513	ZDB-GENE-030616-566
	*SLC1A5*	*slc1a5*	100002129	NM_001190755	ZDB-GENE-070501-4
	*SLC1A6*	*slc1a6*	559270	NM_001109703	ZDB-GENE-071004-45
	*SCL1A7*	*slc1a7a*	100170783	NM_001291344	ZDB-GENE-061009-24
		*slc1a7b*	100463517	NM_001190760	ZDB-GENE-101111-7
	*SLC1A8*	*slc1a8a*	570702	XM_694211	ZDB-GENE-101111-8
		*slc1a8b*	564474	NM_001190816	ZDB-GENE-070912-552
	*SLC1A9*	*slc1a9*	100463516	NM_001190759	ZDB-GENE-101111-9

* Zebrafish *grm2b* has previously been known as *grm7*.

**Table 2:** Zebrafish genes involved in GABA neurotransmission.

Common Name	Human	Zebrafish	Gene ID	RefSeq	ZFIN ID
Na-K-Cl cotransporter 1 (NKCC1)	*SLC12A2*	*slc12a2*	415170	NM_001002080	ZDB-GENE-040625-53
K-Cl cotransporter 2 (KCC1)	*SLC12A5*	*slc12a5*	797331	NM_001302243	ZDB-GENE-120927-3
4-aminobutyrate transaminase	*ABAT*	*abat*	378968	NM_201498	ZDB-GENE-031006-4
Glutamate decarboxylase	*GAD1*	*gad1a*	100329827	XM_005167412	ZDB-GENE-070912-472
		*gad1b*	378441	NM_194419	ZDB-GENE-030909-3
	*GAD2*	*gad2*	550403	NM_001017708	ZDB-GENE-030909-9
Vesicular GABA transporter (VGAT)	*SLC32A*	*slc32a1*	798575	NM_001080701	ZDB-GENE-061201-1
GABA Transporter 1 (GAT1)	*SLC6A1*	*slc6a1a*	692318	NM_001045287	ZDB-GENE-060519-23
		*slc6a1b*	492490	NM_001007362	ZDB-GENE-041114-57
		*slc6a1l*	568985	XM_692346	ZDB-GENE-041210-296
GABA Transporter 3 (GAT3)	*SLC6A11*	*slc6a11a*	558960	NM_001098387	ZDB-GENE-030131-3729
		*slc6a11b*	100150472	XM_001919885	ZDB-GENE-121116-2
Succinic semialdehyde dehydrogenase	*ALDH5A*	*aldh5a1*	565235	NM_001110468	ZDB-GENE-070228-2
GABA Receptors					
GABA_A_ Receptor Subunit α	*GABRA1*	*gabra1*	768183	NM_001077326	ZDB-GENE-061013-194
	*GABRA2*	*gabra2*	100150704	XM_009307207	ZDB-GENE-141216-16
	*GABRA3*	*gabra3*	100538116	XM_009295708	ZDB-GENE-091204-365
	*GABRA4*	-	-	-	-
	*GABRA5*	*gabra5*	799124	XM_001339475	ZDB-GENE-081104-30
	*GABRA6*	*gabra6a*	393704	NM_200731	ZDB-GENE-040426-1692
		*gabra6b*	559693	XM_002667357	ZDB-GENE-080815-1
GABA_A_ Receptor Subunit β	*GABRB1*	*gabrb1*	100331377	XM_002664133	ZDB-GENE-090313-230
	*GABRB2*	*gabrb2*	336252	NM_001024387	ZDB-GENE-030131-8196
		*gabrb2l*	100332196	XM_005174450	ZDB-GENE-111215-5
	*GABRB3*	*gabrb3*	566922	XM_005166079	ZDB-GENE-101102-2
	-	*gabrb4*	566514	XM_005173874XM_017353011	ZDB-GENE-070424-211
GABA_A_ Receptor Subunit γ	*GABRG1*	-	-	-	-
	*GABRG2*	*gabrg2*	553402	NM_001256250	ZDB-GENE-091118-65
	*GABRG3*	*gabrg3*	567057	XM_009302568	ZDB-GENE-070718-5
GABA_A_ Receptor Subunit δ	*GARBD*	*gabrd*	571422	XM_695007	ZDB-GENE-081105-170
GABA_A_ Receptor Subunit π	*GABRP*	*gabrp*	566633	XM_005173293	ZDB-GENE-081028-62
GABA_A_ Receptor Subunit ρ	*GABRR1*	*gabrr1*	568984	NM_001025553	ZDB-GENE-040724-212
	*GABR2*	*gabrr2a*	751659	NM_001045376	ZDB-GENE-060825-164
		*gabrr2b*	569032	XM_692394	ZDB-GENE-041014-174
	*GABR3*	*gabrr3a*	570876	NM_001128760	ZDB-GENE-080722-20
		*gabrr3b*	-	-	ZDB-GENE-131120-131
GABA_A_ Receptor Subunit ζ	*GABRZ*	*gabrz*	561738	NM_001114742	ZDB-GENE-080303-26
GABA_B_ Receptor 1	*GABBR1*	*gabbr1a*	373873	XM_689405	ZDB-GENE-030904-5
		*gabbr1b*	558708	XM_005170102	ZDB-GENE-060503-5
GABA_B_ Receptor 2	*GABBR2*	*gabbr2*	560267	NM_001144043	ZDB-GENE-060503-620

**Table 3:** Zebrafish genes involved in catecholamine neurotransmission.

Common Name	Human	Zebrafish	Gene ID	RefSeq	ZFIN ID
Tyrosine hydroxylase	*TH*	*th*	30384	NM_131149	ZDB-GENE-990621-5
Aromatic amino acid decarboxylase	*AAAD*	*aaad*	406651	NM_213342	ZDB-GENE-040426-2656
Dopamine-β-hydroxylase	*DBH*	*dbh*	30505	NM_001109694	ZDB-GENE-990621-3
Phenylethanolamine-*N*-methyltransferase	*PNMT*	*pnmt*	100332609	XM_002666341	-
Vesicular monoamine transporter 2 (VMAT2)	*SLC18A2*	*slc18a2*	553304	NM_001256225	ZDB-GENE-080514-1
Dopamine transporter (DAT)	*SLC6A3*	*slc6a3*	80787	NM_131755	ZDB-GENE-010316-1
Norepinephrine transporter (NET)	*SLC6A2*	*slc6a2*	565776	XM_689046	ZDB-GENE-110408-4
Catechol-*O*-methyltransferase	*COMT*	*comta*	561372	NM_001030157	ZDB-GENE-050913-117
		*comtb*	565370	NM_001083843	ZDB-GENE-040724-164
Dopamine Receptors	*DRD1*	*drd1a*	792634	XM_017359120	ZDB-GENE-130522-1
		*drd1b*	568126	NM_001135976	ZDB-GENE-070524-2
	*DRD2*	*drd2a*	282557	NM_183068	ZDB-GENE-021119-2
		*drd2b*	378719	NM_197936	ZDB-GENE-030910-2
		*drd2l*	378718	NM_197935	ZDB-GENE-030910-1
	*DRD3*	*drd3*	282554	NM_183067	ZDB-GENE-021119-1
	*DRD4*	*drd4a*	503564	NM_001012616	ZDB-GENE-070112-996
		*drd4b*	503565	NM_001012618	ZDB-GENE-070508-3
	*DRD5*	*drd5a*	100536970	XM_003199767	ZDB-GENE-130522-2
		*drd5b*	-	-	ZDB-GENE-130522-3
Adrenergic Receptors	*ADRA1A*	*adra1aa*	798498	NM_001324454	ZDB-GENE-030131-2831
		*adra1ab*	557259	XM_680297	ZDB-GENE-060503-384
	*ADRA1B*	*adra1ba*	100149100	XM_001921978	ZDB-GENE-120510-1
		*adra1bb*	492486	NM_001007358	ZDB-GENE-041114-51
	*ADRA1D*	*adra1d*	568614	XM_691951	ZDB-GENE-090312-203
	*ADRA2*	*adra2a*	266750	NM_207637	ZDB-GENE-021010-1
	*ADRA2B*	*adra2b*	266751	NM_207638	ZDB-GENE-021010-2
	*ADRA2C*	*adra2c*	266752	NM_207639	ZDB-GENE-021010-3
		*adra2da*	266754	NM_194364	ZDB-GENE-021010-4
		*adra2db*	266755	NM_194365	ZDB-GENE-021010-5
	*ADRB1*	*adrb1*	557194	NM_001128689	ZDB-GENE-081022-145
	*ADRB2*	*adrb2a*	565838	NM_001102652	ZDB-GENE-100414-3
		*adrb2b*	100037315	NM_001089471	ZDB-GENE-070410-32
	*ADRB3*	*adrb3a*	558248	NM_001128335	ZDB-GENE-080917-21
		*adrb3b*	792519	NM_001135134	ZDB-GENE-081022-154

**Table 4:** Zebrafish genes involved in serotonin neurotransmission.

Common Name	Human	Zebrafish	Gene ID	RefSeq	ZFIN ID
Tryptophan hydroxylase	*TPH1*	*tph1a*	352943	NM_178306	ZDB-GENE-030317-1
		*tph1b*	415103	NM_001001843	ZDB-GENE-030805-6
	*TPH2*	*tph2*	407712	NM_001310068	ZDB-GENE-040624-4
		*tph3*/*th2*	414844	NM_001001829	ZDB-GENE-050201-1
Aromatic amino acid decarboxylase	*AAAD*	*aaad*	406651	NM_212827	ZDB-GENE-040426-2656
Monoamine oxidase	*MAO-A*, *MAO-B*	*mao*	404730	NM_001039972	ZDB-GENE-040329-3
Serotonin Transporter (SERT)	*SLC6A4*	*slc6a4a*	664719	NM_001177459	ZDB-GENE-060314-1
		*slc6a4b*	664770	NM_001123321	ZDB-GENE-060314-2
Serotonin Receptors	*HTR1A*	*htr1aa*	100001828	NM_001145766	ZDB-GENE-071203-1
		*htr1ab*	797538	NM_001128709	ZDB-GENE-090409-2
	*HTR1B*	*htr1b*	561647	NM_001145686	ZDB-GENE-081022-141
		*htr1d*	556429	NM_001145686	ZDB-GENE-090409-3
		*htr1fa*	100005344	XM_017357893	ZDB-GENE-081105-125
	*HTR2*	*htr2a*	560808	NM_001044743	ZDB-GENE-070912-500
		*htr2b*	751784	NM_001044743	ZDB-GENE-081022-57
	*HTR2CL1*	*htr2cl1*	100000981	XM_001339004	ZDB-GENE-081104-48
	*HTR2CL2*	*htr2cl2*	798599	XM_001339004	ZDB-GENE-120215-109
	*HTR3*	*htr3a*	571641	XM_009295409	ZDB-GENE-071012-5
		*htr3b*	571632	NM_001126410	ZDB-GENE-071012-4
	*HTR5*	*htr5a*	100038775	NM_001007121	ZDB-GENE-060531-129
	*HTR5-like*	*htr5al*	368475	XM_009297078	ZDB-GENE-030616-574
	*HTR6*	*htr6*	568269	XM_685507	ZDB-GENE-030131-7839
	*HTR7*	*htr7*	562111	NM_178306	ZDB-GENE-130530-666

**Table 5:** Zebrafish genes involved in cholinergic neurotransmission.

Common Name	Human	Zebrafish	Gene ID	RefSeq	ZFIN ID
High-affinity choline transporter	*SLC5A7*	*slc5a7a*	100005589	XM_005159931	ZDB-GENE-090313-273
		*slc5a7b*	-	-	ZDB-GENE-140429-1
Choline acetyltransferase	*CHAT*	*chata*	100170938	NM_001130719	ZDB-GENE-080102-2
		*chatb*	103171573	NM_001291882	ZDB-GENE-140429-2
Vesicular ACh transporter (VAChT)	*SLC18A3*	*slc18a3a*	559347	NM_001077550	ZDB-GENE-060929-990
		*slc18a3b*	394082	NM_201107	ZDB-GENE-040426-1410
Acetylcholinesterase	*ACHE*	*ache*	114549	NM_131846	ZDB-GENE-010906-1
Nicotinic Cholinergic Receptors	*CHRNA1*	*chrna1*	30725	NM_131445	ZDB-GENE-980526-137
	*CHRNA2*	*chrna2a*	678575	NM_001040327	ZDB-GENE-040108-2
		*chrna2b*	568849	XM_692206	ZDB-GENE-041001-99
	*CHRNA3*	*chrna3*	568467	XM_001921279	ZDB-GENE-070822-1
	*CHRNA4*	*chrna4a*	-	-	ZDB-GENE-130530-903
		*chrna4b*	556619	NM_001048063	ZDB-GENE-090505-3
	*CHRNA5*	*chrna5*	550584	NM_001017885	ZDB-GENE-050417-440
	*CHRNA6*	*chrna6*	555747	NM_001042684	ZDB-GENE-090312-91
	*CHRNA7*	*chrna7*	394199	NM_201219	ZDB-GENE-040108-3
	*CHRNA9*	*chrna9*	568807	XM_001920859	ZDB-GENE-090312-63
	*CHRNA10*	*chrna10a*	556507	NM_001044804	ZDB-GENE-060503-725
		*chrna10b*	-	-	ZDB-GENE-130530-624
Muscarinic Cholinergic Receptors	*CHRM1*	*chrm1a*	792708	XM_001332257	ZDB-GENE-090410-9
		*chrm1b*	794658	NM_178301	ZDB-GENE-070705-188
	*CHRM2*	*chrm2a*	352938	NM_178301	ZDB-GENE-030314-1
		*chrm2b*	555516	XM_678041	ZDB-GENE-090410-3
	*CHRM3*	*chrm3a*	571679	XM_695289	ZDB-GENE-090410-4
		*chrm3b*	100149598	XM_001919125	ZDB-GENE-090410-5
	*CHRM4*	*chrm4a*	100150701	XM_001922407	ZDB-GENE-090410-6
		*chrm4b*	-	-	ZDB-GENE-090410-7
	*CHRM5*	*chrm5a*	553978	NM_001020803	ZDB-GENE-080723-32
		*chrm5b*	561491	NM_001030160	ZDB-GENE-041001-169

**Table 6:** Zebrafish genes involved in histamine neurotransmission.

Common Name	Human	Zebrafish	Gene ID	RefSeq	ZFIN ID
l-Histidine decarboxylase	*HDC*	*hdc*	793609	NM_001102593	ZDB-GENE-080102-5
Amine oxidase	*AOC1*	*aoc1*	555401	NM_001077598	ZDB-GENE-061103-112
Histamine *N*-methyltransferase	*HNMT*	*hnmt*	445242	NM_001003636	ZDB-GENE-040801-157
Histamine Receptors	*HRH1*	*hrh1*	735302	NM_001042731	ZDB-GENE-070531-3
	*HRH2*	*hrh2a*	735303	NM_001045338	ZDB-GENE-070531-4
		*hrh2b*	100005590	NM_001109738	ZDB-GENE-070928-20
	*HRH3*	*hrh3*	561773	NM_001025518	ZDB-GENE-040724-204

**Table 7:** Zebrafish genes involved in glycine neurotransmission.

Common Name	Human	Zebrafish	Gene ID	RefSeq	ZFIN ID
Glycine dehydrogenase	*GLDC*	*gldc*	321621	NM_199554	ZDB-GENE-030131-340
Serine hydroxymethyltransferase 1	*SHMT1*	*shmt1*	394021	NM_201046	ZDB-GENE-040426-1558
Serine hydroxymethyltransferase 2	*SMHT2*	*shmt2*	100144628	NM_001123374	ZDB-GENE-071213-1
Inhibitory amino acid transporter	*SLC32A1*	*slc32a1*	798575	NM_001080701	ZDB-GENE-061201-1
Glycine transporter 1	*SLC6A9*	*slc6a9*	494490	NM_001030073	ZDB-GENE-050105-3
Glycine transporter 2	*SLC6A5*	*slc6a5*	494450	NM_001009557	ZDB-GENE-050105-2
Glycine receptor α	*GLRA1*	*glra1*	30676	NM_131402	ZDB-GENE-991117-1
	*GLRA2*	*glra2*	793646	NM_001167899	ZDB-GENE-090407-1
	*GLRA3*	*glra3*	192124	NM_152965	ZDB-GENE-020402-1
	*GLRA4*	*glra4a*	83413	NM_131782	ZDB-GENE-010410-3
		*glra4b*	192125	NM_001202511	ZDB-GENE-020402-2
Glycine receptor β	*GLRB*	*glrba*	83412	NM_131781	ZDB-GENE-010410-2
		*glrbb*	445193	NM_001003587	ZDB-GENE-040801-106

**Table 8:** Reported neurotransmitter levels in control zebrafish brain.

Reference (Age, Sex, and Strain if Known)	Glutamate	GABA	DA	NE	5-HT	ACh
**Adult Zebrafish**						
Panula et al. [16] (Sex, age, and strain unknown)	-	-	2.09 ± 0.42 nmol/g	4.53 ± 0.97 nmol/g	-	-
López Patiño et al. [197] (Male and female 9 ± 1 mpf AB wildtype)	-	-	1.5–2 pg/ug protein	-	-	-
Chatterjee and Gerlai [191] (Male and female 90 dpf AB wildtype)	-	-	4.18 ± 0.28 ng/mg protein	-	-	-
**Embryonic/Larval Zebrafish**						
Wirbisky et al. [193] (Embryos raised at 28.5 °C)	-	-	78.31 ± 2.26 ng/fish (48 hpf) 99.17 ± 6.54 ng/fish (72 hpf)	-	-	-
Tufi et al. [195] (Embryos raised at 26 °C)	9.1 ± 0.5 ng/embryo (48 hpf) 12 ± 0.3 ng/embryo (72 hpf)	1.8 ± 0.03 ng/embryo (48 hpf) 2.2 ± 0.1 ng/embryo (72 hpf)	-	-	7.2 ± 0.01 pg/embryo (48 hpf) 7.1 ± 0.1 pg/embryo (72 hpf)	3.0 ± 0.1 pg/embyro (48 hpf) 4.0 ± 0.1 pg/embyro (72 hpf)
